# Comparing the Efficacy and Safety of Denosumab with Bisphosphonates in Increasing Bone Mineral Density in Patients with Prostate Cancer and Breast Cancer on Antihormonal Treatment

**DOI:** 10.7759/cureus.6401

**Published:** 2019-12-17

**Authors:** Abdul Razaq, Safeera Khan, Junaid Hassan, Bilal Haider Malik, Mahrukh Razaq

**Affiliations:** 1 Urology, DHQ Teaching Hospital, Gujranwala, PAK; 2 Family Medicine, California Institute of Behavioral Neurosciences and Psychology, Fairfield, USA; 3 General Surgery, Mayo Hospital, King Edward Medical University, Lahore, PAK; 4 Internal Medicine, California Institute of Behavioral Neurosciences and Psychology, Fairfield, USA; 5 Obstetrics and Gynecology, Tehsil Headquarter Hospital Kamonki, Gujranwala, PAK

**Keywords:** denosumab, bisphosphonates, osteoporosis, breast cancer, prostate cancer

## Abstract

Osteoporosis is a common condition prevalent in both sexes that can be primary and secondary. Secondary osteoporosis may occur in cancer patients undergoing antihormonal treatment, leading to an increased risk of fractures. Androgen deprivation therapy (ADT) in patients with prostate cancer and aromatase inhibitors (AI) in patients with breast cancer can drastically increase the risk of osteoporosis. Bisphosphonates are one of the key medications in managing these patients and are widely prescribed. A monoclonal antibody called denosumab, which is a relatively new treatment option, is also used in this population group. To conduct a detailed comparison of these groups, we performed a thorough literature search using Pubmed and Google Scholar to extract data in the form of research papers/clinical trials. A total of 18 research papers were extracted using Preferred Reporting Items for Systematic Reviews and Meta-analyses (PRISMA) guidelines and other inclusion and exclusion criteria. Seven of these papers were based on randomized controlled trials (RCTs) comparing denosumab with either placebo or bisphosphonates in patients with breast cancer and prostate cancer. Two meta-analyses comparing the safety and efficacy of both these drugs in this population group were also included. Denosumab was found to significantly increase bone mineral density (BMD) for up to two years and showed better results than bisphosphonates, while both had a comparable safety profile. More trials should be conducted in patients with prostate cancer or breast cancer on ADT or AI therapy, respectively, for longer durations to assess the long-term safety of these drugs in this population.

## Introduction and background

Osteoporosis is a common condition that affects both sexes. It is defined as a disease of the bone characterized by -2.5 standard deviations or less than the mean of bone mineral density (BMD). Primary osteoporosis is more common and is generally age-related, affecting 70-80% of all patients with osteoporosis. Secondary osteoporosis results from secondary conditions like diseases or treatments of diseases (e.g., corticosteroid treatment, anti-hormonal treatment) and can occur at any age. Patients with malignancies, which require antihormonal therapy, like prostate cancer in men and breast cancer in women, may develop bone disease linked to the metastasis or the treatment of the metastasis, such as androgen deprivation therapy (ADT) and anti-estrogen therapy, which can cause bone loss or decreased BMD. Bone loss and associated complications are common conditions in old age that are amplified in cancer patients [1]. Antihormonal therapy for both of these receptor-positive common cancers (i.e., prostate and breast cancers) has proven to be an effective treatment option with great efficacy but also leads to certain side-effects like osteoporosis and decreased BMD, which increases the propensity of fractures in vertebral and weight-bearing joints of the axial skeleton (e.g., the hips) [2].

In the US, prostate cancer and breast cancer are frequently diagnosed in men and women, respectively [3]. They are also common cancers globally, with 900,000 cases of prostate cancer and 1,400,000 cases of breast cancer diagnosed annually [4]. Early detection and appropriate treatment of these malignancies have improved prognosis. Patients with these cancers who are hormone receptor-positive are treated with anti-hormonal therapy, improving their prognosis and reducing recurrence.

Among patients with breast cancer, approximately 75% of receptor-positive cases (estrogen or progesterone) are hormone-sensitive and may, therefore, benefit from anti-hormonal treatment. Aromatase inhibitors (AI) hinder the transition of androgen to estrogen, causing low estrogen levels, resulting in decreased BMD and an increase in the risk of fractures [5]. AIs include serum estrogen receptor modulators (SERMs) and luteinizing hormone-releasing hormone (LHRH) agonists [6].

For prostate cancer, anti-hormonal treatment like ADT is used in hormone-sensitive patients with either localized prostate cancer or advanced- stage prostate cancer with metastasis [7,8]. ADT includes gonadotropin-releasing hormone (GnRH) agonists or GnRH antagonists with or without androgen receptor antagonists and orchiectomy [6]. ADT can increase bone absorption and impair new bone formation, which can ultimately cause decreased BMD, leading to a higher risk of subsequent fractures. Osteoporosis secondary to ADT is rapid and severe and has been found to cause loss of BMD up to 17.3 % greater than controls from six months to one year in one of the studies conducted [9,10]. The occurrence of ADT-induced osteoporosis is higher than osteoporosis in older men or postmenopausal women with twice the incidence as compared to osteoporosis in breast cancer patients on AI therapy [11,12].

One of the mainstays of treatment of osteoporosis is bisphosphonate, which is known to increase BMD and thereby decreasing fracture risks. A newer option is denosumab, which was first approved by the US Food and Drug Administration (FDA) in 2010. Denosumab is a monoclonal antibody subcutaneously administered biannually and has proven beneficial by improving BMD in osteoporotic patients in general, and in patients with prostate cancer and breast cancer who also have bone effects. These bone effects may be due to cancer itself or caused by anti-hormonal treatment [13]. Denosumab can also be used for osteoporosis prevention in castration-resistant prostate cancer, which is usually treated with adjuvant anti-hormonal therapy [8]. It acts by binding to the receptor activator of nuclear factor-kappa B ligand (RANKL), preventing RANK-RANKL binding, thereby causing inhibition of osteoclast activation [13,14].

Our review article will focus on the efficacy and safety of denosumab in comparison to bisphosphonates in managing osteoporosis caused by antihormonal therapy in breast and prostate cancer patients.

## Review

**Figure 1:**
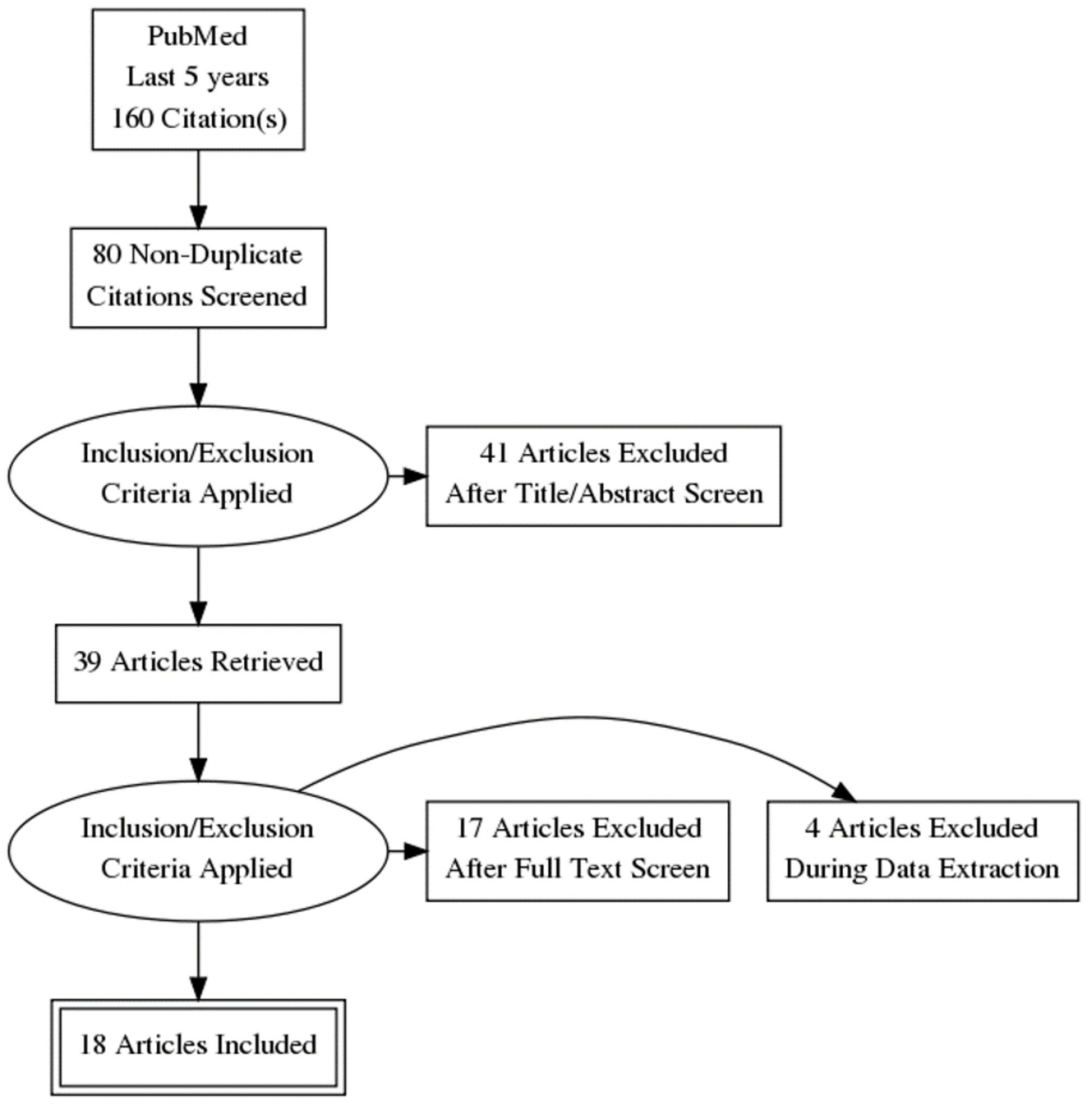
PRISMA diagram showing the selection of data PRISMA: Preferred Reporting Items for Systematic Reviews and Meta-analyses

We conducted a thorough literature search via PubMed and Google Scholar for the relevant published studies. We used "denosumab, bisphosphonates, osteoporosis, Ca (cancer) prostate, Ca breast" as keywords, both separately and in combination with anti-estrogen and anti-androgen therapy. We selected research papers from the past five years. The results of each search term are presented in Table 1. Of these research papers, 80 papers were selected based on the relevance of title and 39 papers were shortlisted after reviewing the abstracts. We applied the inclusion and exclusion criteria, removed duplicate papers, and selected only full-text papers in English. Finally, a total of 18 research papers were included for this review (Figure 1). A few other supportive references were also considered for the introduction and discussion sections.

**Table 1:** Keyword search results by database Ca: cancer

Keywords	Articles found	
	PubMed	Google Scholar
Denosumab	96	10,300
Bisphosphonates	334	19,900
Ca prostate	511	260,000
Ca breast	1,021	474,000
Osteoporosis	773	135,000
Osteoporosis and denosumab	63	7,030
Osteoporosis and bisphosphonates	170	16,400
Osteoporosis, denosumab, and Ca prostate	0	2,560
Osteoporosis, denosumab, and Ca breast	1	3,940
Osteoporosis, bisphosphonates, and Ca prostate	0	5,170
Osteoporosis, bisphosphonates, and Ca breast	4	5,010

Inclusion and exclusion criteria

Randomized clinical trials (RCTs) published in the past five years were selected for the review. These RCTs studied the efficacy of denosumab or bisphosphonates on increasing BMD in patients with prostate cancer or breast cancer undergoing antihormonal therapy. A few meta-analyses comparing both groups of drugs were also included for nonstatistical analysis of these drugs. Clinical trials assessing the safety of these drugs were also considered. Research papers not published in English were excluded. Editorials and non-RCTs were also excluded. Research studies that involved breast cancer or prostate cancer patients with metastasis were not included.

Limitations

There were not many RCTs that compared both of these drugs in prostate and breast cancer. Most of the studies compared one of these drugs with a placebo or studied them alone. There were no studies that assessed the safety profile for a long duration, which was a limitation in comparing both these drugs in terms of safety.

Results

Of the 18 selected research papers, there were seven RCTs using denosumab in breast cancer and prostate cancer [9,15-20]. Three of these RCTs evaluated the role of denosumab in breast cancer [16,17,18], while one evaluated the role of denosumab in prostate cancer [15]. There were two systematic reviews and meta-analyses that compared the role of denosumab with bisphosphonates in both prostate cancer and breast cancer [9,20]. Both meta-analyses included studies published beyond the last five years in this population. One of the studies used denosumab in osteoporosis to evaluate the role of ultrasound in the assessment of the increase in BMD, in comparison to dual-energy X-ray absorptiometry (DXA) [19]. Almost all RCTs used an increase in BMD as an endpoint for efficacy, and fragility fracture or any other serious side effect as an endpoint for safety. Two other clinical trials were included that compared denosumab and bisphosphonates in osteoporotic patients but not specifically in patients with breast cancer and prostate cancer [21,22]. Table 2 presents a selection of studies from the review [9,15-20].

**Table 2:** Selected studies included in the review RCT: randomized controlled trial; Ca: cancer; ADT: androgen deprivation therapy; SERM: selective estrogen receptor modulators; AI: aromatase inhibitors; BMD: bone mineral density; QUS: quantitative ultrasound; DXA: dual-energy X-ray absorptiometry

Study	Location	Study Type	Drugs Used/Patient Group	Result	Conclusion
Joseph et al. [9]	Australia	Systematic review, meta-analysis	Denosumab, bisphosphonates, and SERMs	Bisphosphate increased BMD at the hip joint and femoral neck. SERMs and denosumab also were effective in increasing BMD	Bisphosphonates and denosumab are effective treatments in reducing bone loss and increasing BMD in the lumbar spine, femoral neck, total hip
Doria et al. [15]	Italy, France, Switzerland	RCT	Denosumab and alendronate in patients of Ca prostate taking ADT	Denosumab increased bone turnover markers and decreased bone resorption markers. It significantly increased BMD up to 5.6% compared to alendronate (1.1% after 24 months)	Presently denosumab is the first-line option for osteoporosis and fracture risk reduction in men secondary to hypogonadism due to ADT
Gnant et al. [16]	Austria and Sweden	RCT	Denosumab and placebo in hormone receptor-positive Ca breast patients treated with AI	Denosumab delayed the development time of the first fracture compared to the placebo group. The group taking denosumab had 92 fractures, while the placebo group had 176 fractures	Denosumab 60 mg subcutaneously as adjuvant therapy every 6 months reduces fracture risk in postmenopausal women with Ca breast using AI, given without further toxicity
Nakatsukasa et al. [17]	Japan	RCT	Denosumab in hormone receptor-positive Ca breast patients for 12 months	BMD of the lumbar spine increased by 4.9% and 6.6% at 6 and 12 months, respectively. BMD at the femoral neck was increased bilaterally	Treatment with denosumab twice a year increases BMD in Japanese women with Ca breast who were receiving AI treatment
Nakatsukasa et al. [18]	Japan	RCT	Denosumab in hormone receptor-positive Ca breast patients for 24 months (secondary follow-up study)	Lumbar spine BMD increased by 5.9 % (18 months) and 7.0% (2 years). BMD of the femoral neck also increased. No serious adverse effects like osteonecrosis of jaw or hypocalcemia occurred	Denosumab twice a year increased BMD in Japanese women with Ca Breast receiving adjuvant AI therapy for up to 2 years
Catalano et al. [19]	Italy	RCT	Denosumab	Denosumab group had improved QUS and DXA measurements at 24 months. Reduced bone markers detected at 12 and 24 months compared to baseline	Denosumab preserves bone health. Phalangeal QUS may be considered in the follow-up AI-treated Ca breast women receiving denosumab
Galvano et al. [20]	Italy	Systematic review, meta-analysis	Denosumab	At 24 months, denosumab showed a BMD increase at the lumbar spine, total hip, femoral neck, distal third radius	Denosumab is an effective and safe treatment for the prevention of vertebral and femoral fragility fractures

Discussion

Prostate cancer is one of the more common cancers in men worldwide [23]. ADT causes a decrease in BMD, which significantly increases the risk for fractures both statistically and clinically, leading to increased morbidity and mortality [9]. Similarly, an increase in BMD will reduce fracture risk. In the vulnerable patients who have a propensity to develop osteoporosis and a greater likelihood of fragility fractures like the ones treated with ADT, risk assessment is important. The fracture risk assessment tool (FRAX) can estimate the fracture risk using patient characteristics [24]. BMD is usually assessed by DXA scan [24], and this can be used with the FRAX tool to assess the risk for fragility fractures in the population at risk.

Mechanism of Action of Denosumab and Bisphosphonates

**Figure 2:**
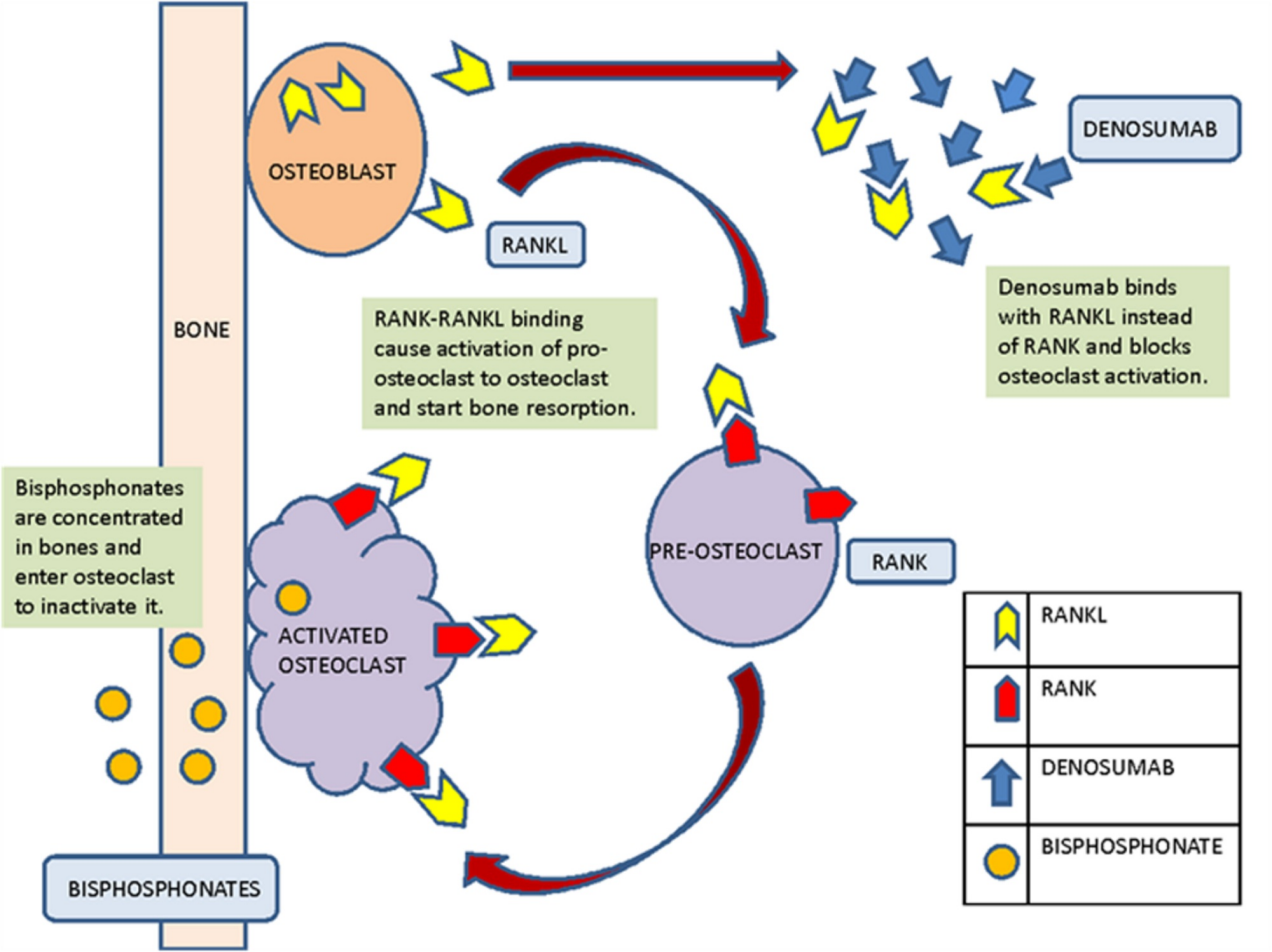
Mechanism of action of denosumab and bisphosphonates on the bone RANKL: receptor activator of nuclear factor-kappa B ligand; RANK: receptor activator of nuclear factor-κappa B (RANK)

Bone normally undergoes constant remodeling throughout life to maintain proper shape and growth. This process involves both osteoblasts and osteoclasts. Bone density gradually decreases with increasing age, but it may also decline because of diseases like prostate cancer and breast cancer. These diseases themselves affect bone tissue, and their treatment (antihormonal therapy) also adversely affects bone, leading to secondary osteoporosis [25]. Normally, estrogen has a key role in maintaining bone density in healthy postmenopausal women; estrogen acts on both osteoblasts and osteoclasts to decrease bone turnover by suppressing bone remodeling action [26]. Antihormonal therapy, whether it is ADT or AI therapy, ultimately decreases estrogen activity [15,27]. Figure 2 illustrates the mechanism of action of denosumab and bisphosphonates.

Osteoblasts, along with bone production, also produce RANKL, which, in turn, binds to the RANK receptors on osteoclasts. This RANKL-RANK binding then activates the osteoclasts, and they start resorbing the bone. This normal regulatory process may contribute to bone loss, which is exacerbated by the disease itself or the treatment of the disease, such as antihormonal therapy such as ADT or AI therapy.

Denosumab, a highly specific inhibitor of RANKL, is a monoclonal antibody that prevents this RANKL-RANK binding by binding itself with RANKL and disrupting the pathway activating the osteoclasts. It acts as a regulator of osteoclastic bone resorption and is a strong bone-resorbing cytokine [29,30]. This will ultimately prevent bone loss; therefore, this drug is used to prevent osteoporosis in both men and women with prostate cancer and breast cancer, respectively, along with their use in primary osteoporosis [30].

Another group of drugs used prior to the development of denosumab for both primary and secondary osteoporosis was bisphosphonates. Bisphosphonates are concentrated directly in bone and can enter the osteoclast where it causes an acidic medium in lacunae after activation [31]. Once internalized, they are toxic for the osteoclast. They either inactivate or trigger apoptosis of the osteoclast depending on whether they are nitrogenous or non-nitrogenous bisphosphonates. They either cause apoptosis directly or inhibit the enzyme farnesyl pyrophosphate synthase [31].

Many bisphosphonates have long been in use for osteoporosis, such as alendronate (oral), risedronate (oral), and zoledronic acid (intravenous). Risedronate is taken daily, weekly or monthly in 5-mg, 35-mg, and 150-mg doses, respectively. Alendronate is usually taken 10 mg once daily or 70 mg weekly. Zoledronic acid is given in infusion form 5 mg intravenously over more than five minutes every one to two years. Denosumab, on the other hand, is given as a 40-mg or 60-mg injection subcutaneously once every six months [9].

In terms of patient adherence and compliance, oral bisphosphonates may not be very convenient due to their frequent dosage, drug interactions, and the requirements like the ingestion of large amounts of water with it. Also, there is a requirement that the patient should not lie down for at least an hour or until the next meal. Injectable bisphosphonates may be preferred by patients as compared to oral forms because of the longer duration of action despite being invasive or inconvenient due to infusion administration [9].

Denosumab, on the other hand, is minimally invasive as compared to injectable bisphosphonates due to its subcutaneous route of administration. Denosumab also has a longer duration of action, offering six months of protection [29]. These characteristics may make it an attractive option for patients and may increase compliance.

Efficacy of Both Drugs in Prostate Cancer and Breast Cancer

Patients with hormone-sensitive nonmetastatic prostate cancer and breast cancer treated with antihormonal therapy (ADT for prostate cancer, AI for breast cancer) are at high risk for osteoporosis and fragility fractures. This cumulatively creates increased comorbidity and risk of mortality in these patients.

Several recent trials have assessed the efficacy of bisphosphonates compared with denosumab in prostate and breast cancer patients. Gnant et al. conducted a phase-3 double-blind RCT (ABCSG-18) in hormone-sensitive postmenopausal breast cancer patients comparing the efficacy of 60-mg denosumab with placebo [16]. They found that the denosumab group had delayed the development of a clinical fracture. The postmenopausal group, which was already a high-risk group for developing osteoporosis, had an increased chance of decreasing their BMD on AI therapy. The postmenopausal group had a significant increase in the development of clinical fracture, indicating the better efficacy of denosumab in increasing BMD and delaying fracture [16].

The effect of denosumab on increasing BMD was evaluated in a prospective study in Japanese women over 12 months, which found that 60- mg subcutaneous denosumab increased BMD in postmenopausal women who were on AI therapy for breast cancer. This increase in BMD was observed at both the lumbar spine and neck of the femur, bilaterally. BMD at the lumbar spine increased up to 4.9% at six months and 6.6% at 12 months, whereas the BMD at the neck of the femur also significantly increased [18]. This study was extended as a prospective second trial to study the increase in BMD from baseline for up to 24 months. At 18 and 24 months, BMD increased up to 5.9% and 7.0% at the lumbar spine, respectively. The study concluded that the administration of denosumab as an adjuvant therapy continuously increased BMD for up to 24 months [17].

A prospective 24-month observational study by Doria et al. compared the effect of denosumab 60 mg subcutaneously with alendronate 70 mg once weekly in prostate cancer patients on ADT [15]. Both denosumab and alendronate improved BMD, but denosumab showed significantly better results (p = <0.001) [15]. Denosumab demonstrated an increase in BMD up to 5.6% at the lumbar spine after 24 months, while alendronate increased BMD by up to 1.1%. This superiority of the denosumab group was further strengthened by the result that there were fewer fractures among the patients taking denosumab than in those taking alendronate. However, this difference was not found to be significant (p = 0.10) [15].

A meta-analysis by Galvano et al. compared the effect of denosumab on BMD with the placebo group in both prostate cancer and breast cancer at 24 and 36 months. The study showed that denosumab reduces BMD loss even up to three years and decreases the incidence of new vertebral fractures at two to three years [20]. The meta-analysis also considered an increase in BMD at the lumbar spine, total hip, head of the femur, and distal radius and showed that denosumab not only reduced bone loss but also increased the BMD, ultimately reducing osteoporosis and risk of subsequent fracture [20]. Another meta-analysis by Joseph et al. showed that both denosumab and bisphosphonates are effective treatment options to increase BMD in prostate cancer patients on ADT [9].

A study assessing the role of qualitative ultrasound (QUS) and DXA in assessing bone health in patients treated with denosumab compared with a control group showed that with denosumab, QUS and DXA measurements were significantly improved (p = <0.05) after two years and bone-turnover markers (carboxy-terminal telopeptide and bone-specific alkaline phosphatase) were reduced at one and two years. Denosumab preserved bone health according to QUS and DXA findings. A study by Catalano et al. concluded that inexpensive and phalangeal QUS could be used as a follow-up tool in all breast cancer patients receiving denosumab [19].

Doria et al. found that denosumab increased BMD in the axial skeleton in men with osteoporosis. They also noted a decrease in bone turnover as seen by a reduction in bone-turnover markers for bone resorption and, ultimately, a reduction in new vertebral fractures [15]. 

Comparing Adverse Effects and Safety Profiles of Both Groups

As patients with breast cancer and prostate cancer need long-term antihormonal therapy, the risk of osteoporosis is increased because of the nature of the treatments and the age of the patients in which these diseases occur. These patients may have bone-related adverse effects because of the disease or its treatment [13]. So, adjuvant therapy to prevent osteoporosis and increase BMD also needs to be given for a long time. This long duration of denosumab and bisphosphonate therapy use in this vulnerable group should be assessed and compared in terms of safety and adverse effects.

Bisphosphonates are commonly used medications and have a generally favorable safety profile, but their long-term use may cause certain adverse effects. Common adverse effects of bisphosphonates include gastrointestinal intolerance, gastritis, osteonecrosis of the jaw, chronic musculoskeletal pain, atypical femur fractures, atrial fibrillation, and esophageal cancer [32]. Intravenous bisphosphonates may also cause renal toxicity [21].

Common adverse effects associated with denosumab are hypocalcemia, shortness of breath, diarrhea, musculoskeletal pain, cellulitis, and infection. Relatively less common adverse effects are hypophosphatemia, osteonecrosis of the jaw, and skin rash [33].

A study by Gnant et al. showed that denosumab could be administered safely in breast cancer patients receiving adjuvant AI therapy without any new or additional adverse effects. By weighing the benefits and risks, the main adverse effect of antihormonal therapy (i.e., osteoporosis) can be reduced, thus making denosumab a valid option [16]. Nakatsukasa et al. reported no serious hypocalcemic events exceeding grade 2 and no osteonecrosis of the jaw among 102 postmenopausal women with breast cancer on AI therapy, which indicated denosumab’s relatively favorable safety profile for one year [18]. Nakatsukasa et al. also studied denosumab in the same population for two years and found no episodes of hypocalcemia exceeding grade 2, osteonecrosis of the jaw, or atypical femoral fractures, providing evidence of a favorable safety profile for up to two years [17].

There are not many safety comparison studies for these two drugs in our population group. One study by Choi et al. compared the safety profiles of denosumab and zoledronic acid in osteoporosis, irrespective of the cause. They found that denosumab had a similar safety profile to zoledronic acid without any additional adverse effects. They also reported a comparable result in terms of safety and efficacy for the considered outcomes of any serious infection, cardiovascular disease, and fractures after one year of use. However, the study did not consider breast cancer or prostate cancer patients on antihormonal therapy; therefore, the results cannot be completely translated for our patient population [34].

Intravenous bisphosphonates like zoledronic acid need to be administered as an infusion, thus requiring an intravenous line. Bisphosphonates are nephrotoxic and require regular monitoring of renal functions, which may prompt the need for dosage adjustment or discontinuation in case of renal compromise. This issue is even more important as the population group affected by prostate cancer is mostly elderly and may already have renal impairment either due to obstruction or other comorbidities. Denosumab is, therefore, an appropriate option given the lack of renal effects and its twice-yearly subcutaneous injection without the need to monitor to the same degree as a zoledronic acid infusion [15,35]. For oral bisphosphonates, patients should not lie down for an hour after taking them; therefore, denosumab can be a good option for patients who are unable to sit or stand. Denosumab can be a preferable option in patients who are unable to tolerate bisphosphonates despite bisphosphonates being more cost-effective. 

A meta-analysis of nine clinical studies comparing the safety and efficacy of denosumab with bisphosphonates in reducing fracture risk showed no significant difference in efficacy and safety after one to two years of their use, even with a relatively greater increase in BMD by denosumab compared to bisphosphonate [36]. A few other safety studies in other populations also showed a similar safety profile. One such study compared a cohort of rheumatoid arthritis patients using denosumab as an adjuvant therapy with disease-modifying antirheumatic drugs (DMARDs) to zoledronic acid as an adjuvant with DMARDs [22]. This study reported a similar rate of hospitalized infections in both groups [22]. Choi et al. also reported that, in patients older than 50 years with osteoporosis, there was no additional risk of developing serious infections or cardiovascular diseases like myocardial infarction, stroke, or heart failure with the use of denosumab compared to zoledronic acid after one year of use [34].

Almost all safety studies of these drugs were conducted over one to two years; therefore, more studies are needed to establish their safety in our population group for a longer duration.

## Conclusions

Osteoporosis secondary to antihormonal therapy usually is treated with bisphosphonates and denosumab in addition to calcium and vitamin D. Our review focused on the comparison of these two drugs in our patient population in terms of efficacy and safety in patients suffering from prostate cancer and breast cancer. Bisphosphonates and denosumab both increased BMD and reduced the risk of serious fractures in this high-risk population and maintained it consistently, even up to two years. Denosumab increased BMD more than bisphosphonate and can be used in patients who cannot tolerate bisphosphonates due to their side effects and dose regimen. In terms of safety, denosumab has a somewhat similar safety profile as bisphosphonates and maybe a better, safer option in patients predisposed to renal compromise. These effects were observed in studies that mostly lasted for up to two to 2.5 years and, therefore, may not accurately reproduce the effects if used for a longer period. There is a need to study their effects specifically in this group for a longer duration for a better insight into their safety profile, especially in patients with other comorbidities.
